# Functional near infrared spectroscopy (fNIRS) to assess cognitive function in infants in rural Africa.

**DOI:** 10.1038/srep04740

**Published:** 2014-04-22

**Authors:** Sarah Lloyd-Fox, M. Papademetriou, M. K. Darboe, N. L. Everdell, R. Wegmuller, A. M. Prentice, S. E. Moore, C. E. Elwell

**Affiliations:** 1 Centre for Brain and Cognitive Development, Birkbeck, University of London; 2 Department of Medical Physics and Bioengineering, University College London; 3 Medical Research Council (MRC) International Nutrition Group, MRC Keneba, MRC Unit, The Gambia; 4 MRC International Nutrition Group, London School of Hygiene & Tropical Medicine, London, UK

## Abstract

Cortical mapping of cognitive function during infancy is poorly understood in low-income countries due to the lack of transportable neuroimaging methods. We have successfully piloted functional near infrared spectroscopy (fNIRS) as a neuroimaging tool in rural Gambia. Four-to-eight month old infants watched videos of Gambian adults perform social movements, while haemodynamic responses were recorded using fNIRS. We found distinct regions of the posterior superior temporal and inferior frontal cortex that evidenced either visual-social activation or vocally selective activation (vocal > non-vocal). The patterns of selective cortical activation in Gambian infants replicated those observed within similar aged infants in the UK. These are the first reported data on the measurement of localized functional brain activity in young infants in Africa and demonstrate the potential that fNIRS offers for field-based neuroimaging research of cognitive function in resource-poor rural communities.

Over the last decade, advances in neuroimaging technology and software have opened a new avenue for research of the developing human brain, allowing us to investigate questions that until recently would have seemed impossible with existing behavioural methods. Several neuroimaging methods are available for the study of infants, the relative attributes of which are outlined below. Firstly, different methods are designed to detect different correlates of brain activation; either direct activation related to electrical activity of the brain (e.g. EEG, magnetoelectroencephalography (MEG)) or the consequent haemodynamic response (e.g. positron emission tomography (PET), functional magnetic resonance imaging, (fMRI), functional near infrared spectroscopy (fNIRS)). Secondly, many of these neuroimaging techniques, which are well established in adults, have limiting factors restricting or preventing their use in infants. PET requires the use of radioisotopes, whilst magnetic resonance imaging MRI and MEG require the participant to remain very still, usually swaddled or restrained. There has been some infant research published using these techniques^1^,^2^,^3^,^4^,^5^,^6^, however this work has generally been restricted to the study of sleeping, sedated or very young infants. For many years, the primary choice for functional imaging in awake infants has been EEG, a non-invasive technique with high temporal resolution but relatively poor spatial resolution. The advent of the new technology, fNIRS, was a welcome addition to a limited choice of neuroimaging methods suitable for use in infants. We believe that fNIRS provides an essential bridge between our current understanding of cortical activity in the developing brain and our knowledge of adult human brain function. It can be widely adopted due to its relatively low cost, ease of use with infants, capacity for more specific spatial localization with respect to EEG, and suitability for use in naturalistic settings^7^,^8^. Though the depth resolution of fNIRS is dependent on the age of the infant and the optical properties of the tissue^9^ and offers lower spatial resolution relative to fMRI, it is similar in that it measures haemodynamic responses to neuronal activation. Research from adults has shown a high degree of correlation between simultaneous recordings of haemodynamic responses with fNIRS and fMRI^10^,^11^. Thus, the data acquired from fNIRS can complement the high spatial resolution of function and anatomy data obtained with MRI. The use of NIRS to study functional brain activation in infants is a rapidly increasing research area^8^,^12^,^13^ and, since the first in 1998, the number of published fNIRS infant studies is now close to 100. The technique has been used to address developmental topics such as; object processing^14^,^15^, social communication^16^,^17^, human action processing^18^,^19^, face processing^20^ and developmental disorders such as autism, both in infants^21^,^22^ and children^23^.

A recent shift in the use of neuroimaging has been towards the study of the infant brain in situations where this development may be compromised in some way. These may include the impact of acute brain injury in early infancy, chronic conditions (such as cerebral palsy), genetically related conditions (such as Williams or Downs Syndrome), developmental disorders (such as autism or ADHD) and the impact of environmental factors (such as poverty and nutrition). The use of neuroimaging methods for the study of atypical infant development may provide information that to date has been unavailable with existing behavioural paradigms. For example, whilst behavioural measures have been unable to distinguish between infants with low and high-risk of developing autism (defined by a familial diagnosis) before the first year of life, several recent EEG and fNIRS studies have identified differences in brain function in younger infants^21^,^22^,^24^,^25^,^26^,^27^,^28^. Recent work on the relationship between family socio-economic status (SES) and infant brain development has evidenced atypical neural activity using EEG in six to nine month old infants with low SES living in the UK, highlighting the importance of the early-life environment on brain development^29^.

One major factor contributing to early environmental impact is nutrition. Approximately 165 million children worldwide are under nourished and stunted^30^, the majority of whom live in Sub-Saharan Africa or South Asia. Those who are most susceptible to stunting (chronic undernutrition defined as below minus two standard deviations from the median WHO child growth standards for height for age) tend to be the poorest and/or those living in a rural area (UNICEF, Global Nutrition Database, 2012). Nutritional status is influenced by three broad factors, health, care and food^30^. From a nutritional perspective the most important time in our development to be well nourished is during the first 1000 days, which includes pregnancy and the first two years of life. Under nutrition occurs in a linear fashion during this period and catch-up growth in later childhood is minimal^31^.

Nutrients and growth factors affect the growth of the brain during prenatal and early postnatal life. Animal studies have shown that malnutrition can cause decreases in brain volume; myelination; number of neurons, synapses, dendrites and reactive zones (for review see^32^). The relative effects of under dosage of different nutrients (such as protein-energy, zinc, iron and long-chain polyunsaturated fatty acids) on cognitive development are an ongoing concern^32^,^33^. Different nutrients may impact on human brain development in different ways. For example, the cortex and hippocampus appear to be particularly vulnerable to a reduction in protein-energy levels^34^ while iron is essential for myelination^35^. Brain and nervous system development in human infants during the first 1000 days is critical, and deficits in nutrition during this time can have a deep impact on cognitive development^36^,^37^,^38^. Recent pooled analysis from longitudinal cohorts in Brazil, Guatemala, India, the Philippines and South Africa, has shown that early recovery of weight gain within the first two years of life is an important predictor of outcome at school^39^. If the recovery of weight gain and improvement in environmental factors occurs in childhood - but after the first two years of life - the impact of early under nutrition has far reaching effects and is still seen in physical growth and cognitive function in adolescence. Therefore, it is paramount that intervention to prevent growth faltering reaches infants from as early an age as possible. Indeed, the broader implications of under nutrition in infancy have been shown in recent research evidencing lifelong effects on adult health, which in turn effect social and economical development within the country^39^,^40^,^41^.

Objective measures of brain function within the first few months and years of life could be used to assess how the timing and nature of pre and postnatal nutritional insults impact on cognitive development, and to inform and evaluate interventional strategies. To date, due to methodological constraints, the majority of work which has assessed the impact of under nutrition on early brain development in low-income countries has been undertaken using behavioural measures^42^. Although useful, these tests can only detect the effects of nutritional deficiencies once they reach the point of observable behaviour, thus reducing the possibility of targeted early intervention strategies. There are also issues relating to the implementation, cultural adaptation and standardisation of behavioural tests for use across different communities in global health studies. Cortical mapping of cognitive function during infancy is poorly understood in low-income countries. The high cost and low portability of neuroimaging methods such as MRI has excluded their use in resource poor settings and field-based research. fNIRS potentially provides an elegant solution to bridge this methodological gap. The technique has long been heralded as a portable, low cost and relatively easy to administer method. Yet to date it has primarily been utilised in high-income easily accessible populations in Europe, North America and Japan, although we have recently used fNIRS to investigate the cerebral haemodynamics in adult patients with cerebral malaria in India^43^. The aim of the current work was to investigate the efficacy of transporting an fNIRS system to rural Gambia and using it to acquire functional brain imaging data in local infants: with a secondary goal of assessing cortical activation in response to social cues in Gambian infants. We use the term ‘social’ in this paper in the broadest sense, i.e. that they are human generated cues, either visual or auditory, which originate from conspecifics. This does not necessarily imply that these cues are intended to be communicative.

Participants were recruited from the West Kiang District, an area of predominantly subsistence farming, which is 145 km inland from the capital of The Gambia. The field station where the study took place is reached by 4x4 vehicles on unmade roads and located within a remote inland village, Keneba. Due to its isolation the field station in the village maintains all facilities necessary for research and clinical care (generator powered electricity, water supplied by bore hole, satellite communication). The primary research objective of the field station is to study the impact of under nutrition and provide interventions to promote healthy growth in the population. A combination of prenatal growth retardation, poor-quality and often contaminated foods and high levels of infection cause moderate to severe growth faltering in height and weight gain from around 3 months of age in the local population^44^,^45^,^46^. The long-term aim of our contribution to this research is to establish fNIRS as an assessment tool for the investigation of the impact of under nutrition on cognitive development in infants in regions such as Keneba.

## Results

**Figure 1:**
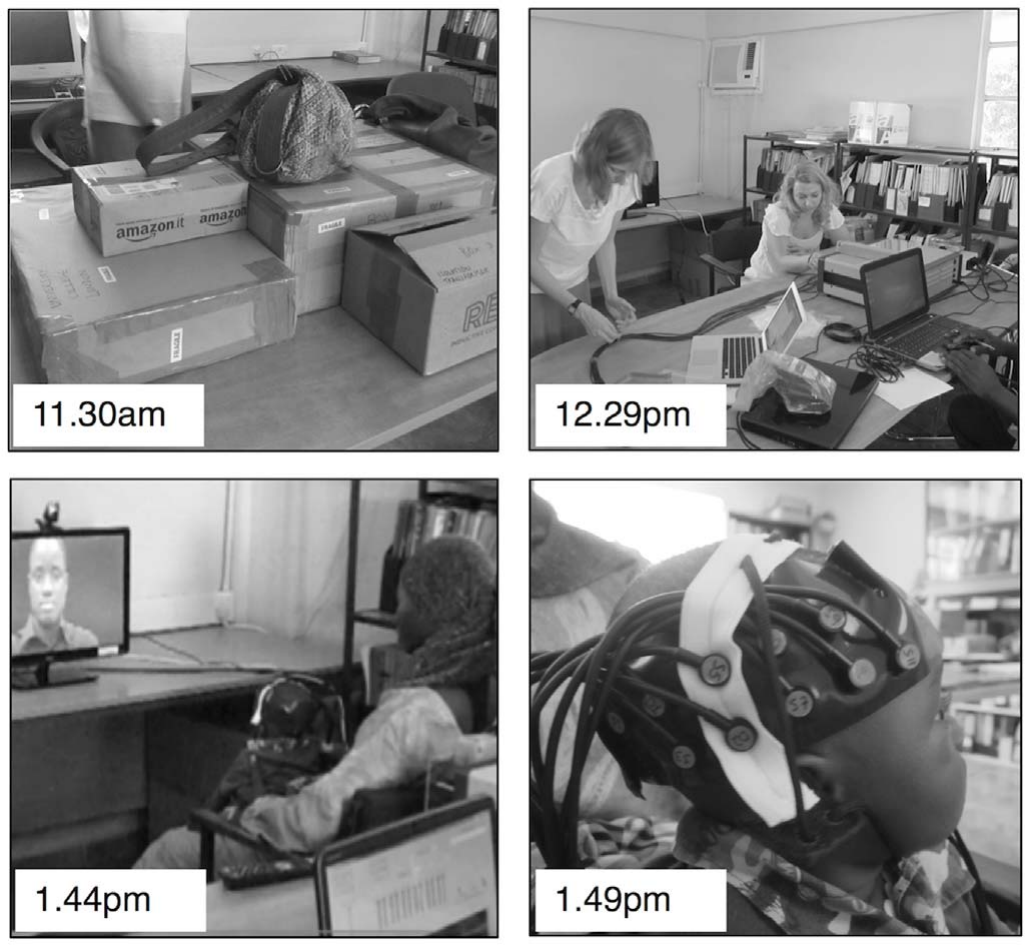
A photo timeline of the setup of the NIRS equipment and first infant to take part in the study at the MRC Field Station in Keneba.

We have successfully performed the first functional brain imaging study in infants in Africa. With the use of fNIRS we were able to measure brain responses to social cues in infants living in a remote rural location in The Gambia. In support of the ease of use of the equipment we were able to setup, train a field worker and run our first study within 2.5 hours of arrival of the fNIRS system (see Figure 1).

**Figure 2:**
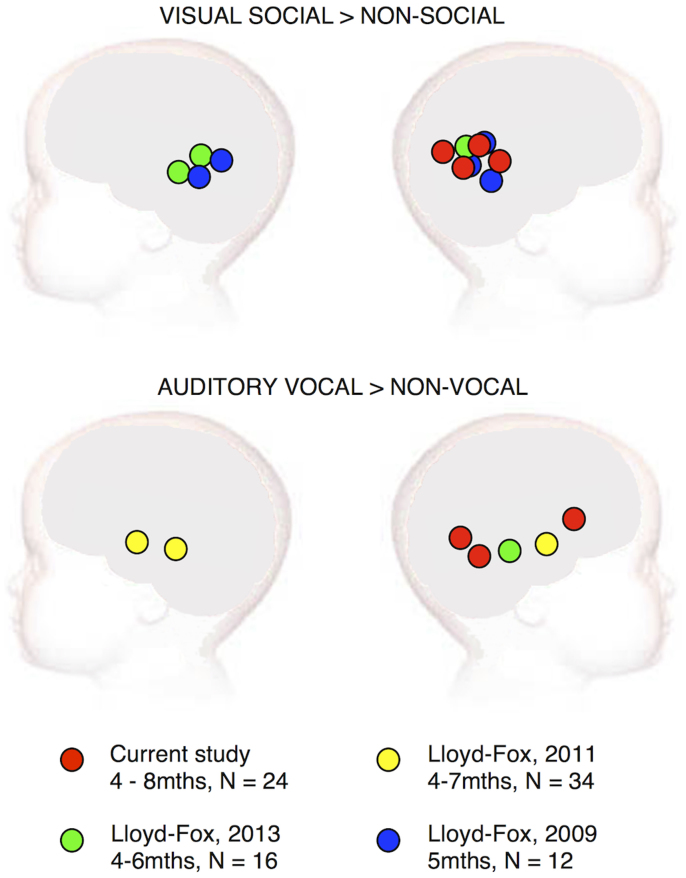
An overview of the anatomical location of the visual social responses and auditory vocally selective (vocal > non-vocal) responses in the current study in relation to three previous publications using the same stimuli. The location of activation is similar across all four groups of 4–8 month old infants from the UK and the Gambia. Note that the Gambia data was measured from the right hemisphere only (figure designed by S. Lloyd-Fox).

The findings reveal that the infants' cortical responses are in concordance with previous cohorts of infants in the UK, revealing localized patterns of activation in regions of the posterior superior temporal and inferior frontal cortex to the visual and auditory social stimuli (see Figure 2 and Supplementary data for figures of the haemodynamic time courses for each condition). Note that while previous work used fNIRS measurement channels over both hemispheres, the current work was restricted to measuring the right hemisphere as the available funding restricted us to building a fNIRS system with a limited number of channels.

### Visual Social Condition

**Figure 3:**
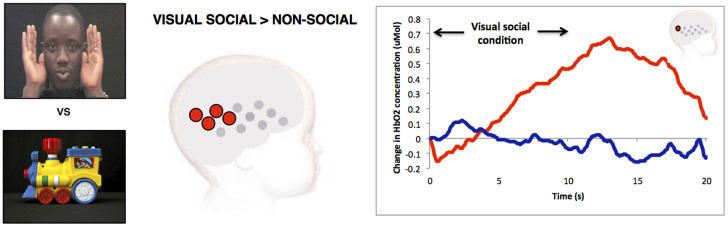
The visual social stimuli (left panel), the location of the significant group haemodynamic responses (visual social > visual non-social) on an average five to six month old head (middle panel) and an example of the haemodynamic response to the visual social and visual non-social conditions for channel 12 (right panel). The significant responses are illustrated in red (HbO_2_) and blue (HHb).

To assess the responses to the visual social stimuli the experimental condition with no sound (visual only) was analysed relative to the non-social visual baseline (t-test, two-tailed). This analysis revealed significant haemodynamic increases in HbO_2_ centred over the posterior area of the arrays (see Figure 3), corresponding to the posterior STS region of the cortex. The response was evident in channels 9, 11, 12, 17 & 18 (channel 9: t = 2.68, p = 0.013; channel 11: t = 3.39, p = 0.0025; channel 12: t = 3.88, p = 0.0008; channel 17: t = 7.49, p = 0.002; channel 18: t = 4.97, p = 0.038). This analysis did not reveal any significant decreases in HHb. The time course for channel 12 is illustrated in Figure 3.

### Auditory Vocal and Non-vocal Conditions

**Figure 4:**
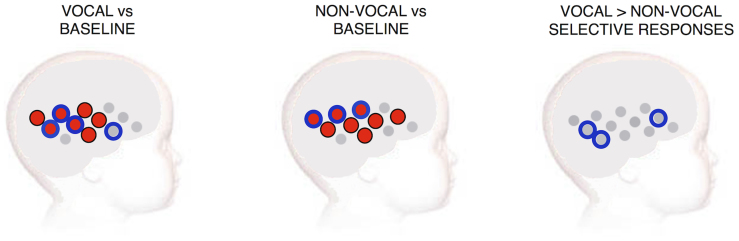
The group haemodynamic responses for the three auditory contrasts: vocal vs silence (left panel), non-vocal vs silence (middle panel) and vocal > non-vocal (right panel) on an average five to six month old head. The significant responses are illustrated in red (HbO_2_) and blue (HHb).

Initially statistical analyses of vocal and non-vocal auditory responses compared with silence (visual social only) were conducted (see Figure 4). This analysis revealed widespread activation in both HbO_2_ and HHb. For both conditions the responses were centred over the temporal cortex. Paired sample channel-by-channel t-tests (two-tailed) were performed to assess the presence of vocal and non-vocal selective activation. This analysis revealed a greater hemodynamic response to the vocal condition relative to the non-vocal condition in channels 2, 10 & 11, centered over the mid-portion of the superior temporal sulcus (STS) region and the inferior frontal cortex (channel 2: t = 2.087, p = 0.048; channel 10: t = 2.15, p = 0.04; channel 11: t = 2.29, p = 0.031; HHb). There were no non-vocal selective responses.

## Discussion

We have successfully performed the first functional brain imaging study in infants in Africa. Furthermore, to our knowledge this is the first study in the world to use a neuroimaging method to study functional brain responses in a field based developmental research study in a remote rural location. We assert that fundamentally this work was only achievable due to the choice of neuroimaging method: fNIRS. Our findings support the portability (transported in a 4x4 vehicle on unmade roads); ease of use (a typical length of study from when the infant entered the room to when it was complete was 20 minutes, and approximately 40 infants visited over 8 days) and suitability for use in naturalistic settings (not dedicated for cognitive assessment measures) of the technology. The technology has the potential to be optimized for even more rural settings with battery-operated systems that could be entirely field based rather than within one centralized location. Furthermore, the technology was well received and tolerated by the field workers, parents and infants. The field workers helped to run the studies alongside the experienced team and with further training the local team would have the expertise necessary to run this work alone.

With the use of fNIRS we were able to measure brain responses to social cues in infants living in a remote rural location in The Gambia. Furthermore, these findings largely replicated previous work conducted by our group in the UK^1^,^22^,^47^,^48^ and previous infant work elsewhere^49^,^50^ (see Figure 2). Interestingly, the specificity of the responses of the Gambian infants to the visual social vs non-social stimuli was the same as the infants in the UK, despite substantial differences in their socioeconomic, cultural and geographic environment. Furthermore, though the human stimuli were re-filmed with a Gambian adult, the baseline condition contained images that may have been unfamiliar to these infants (i.e. helicopter, train). Due to this, we may have predicted that the activation to the visual social stimuli in contrast to baseline would have been diminished or altered, however this does not appear to be the case in the current work. In this study we use the term “social” in its broadest sense, to refer to “human-generated” visual or auditory cues. The social visual stimulus were selected to ensure compatibility with previous studies^48^,^51^, however, as in previous work we acknowledge that the cortical responses to the visual stimuli may be a response to the perception of biological motion, rather than the social relevance of the stimuli. The cortical activation seen in response to the vocal vs non-vocal contrast was broadly similar in the Gambian infants compared with previous studies performed in Europe^22^,^47^,^49^. Though the location of the activation did not overlap to the extent of the response to the visual-social response, this may not be surprising given that the same stimulus contrast in adults^52^ causes activation along the full extent of the middle and superior temporal region (which is the region activated in the current work and previous infant work^22^,^47^,^49^; see^47^ for a summary figure of the infant and adult overlapping patterns of activation). In contrast to previous findings, we also found vocal-selective activation in a channel over the inferior frontal region of the cortex, suggesting that these stimuli may have caused a broader pattern of activation in the Gambian infants. Interestingly, in the current study we did not find selective activation for the non-vocal vs vocal contrast. In previous work this selective response was found in 4–6 mth olds^47^,^49^ but not in 7 mth olds^49^, therefore given that our participants were 4–8 mth olds it is possible that this response is moderated by age. These finding should be addressed further in future work.

Recent work has highlighted the potential for fNIRS data to compliment the findings from other assessments and provide greater sensitivity and specificity of our understanding of cognition. An fNIRS study with infants under 6 months of age^18^ has identified a linear relationship between the specificity of infants' brain responses and their own motor development. This would suggest that action production and action perception are closely linked from an early age. A recent study with 9 month olds^53^ used fNIRS to investigate the neural underpinning of behavioural responses to colour priming. They found that a change in looking behaviour during an object function manipulation task was associated with activation in the anterior temporal cortex, thought to reflect a cortical signature of object individuation. However, importantly, they also found increased activation in the posterior temporal cortex following exposure to a change in object function or motion despite an absence of a behavioural response to the latter. This recent work highlights the potential this technique offers for going beyond behavioural evidence with objective markers of brain function.

A major advantage of fNIRS is that it can provide a measure of spatial specificity of neural deficit or altered cognitive function, which could inform targeted interventions. A study with adults^54^ revealed alterations in the shape of the haemodynamic response to visual stimulation in migraine sufferers relative to a control group, which was normalised with intervention using precision spectral filters. fNIRS has recently identified atypical or absent cortical responses in infants at high risk for developing autism due to a familial diagnosis^21^,^22^. Lloyd-Fox and colleagues^22^ used the same paradigm as the current study and found diminished activation in response to both the visual and auditory social cues in infants at high risk of developing autism when compared with age matched low risk infants. These differences in response were seen as early as 4–6 months of age and, in the absence of early behavioural markers. These findings highlight the sensitivity of fNIRS as a tool to provide an early biomarker of atypical development and to identify the physiological mechanisms behind atypical brain function. Recent fNIRS research in adults has evidenced changes in activation in response to nutritional intervention (for a review see^55^), but to date there have been no such studies in infants. There is therefore a clear rationale for using fNIRS to provide early predictors of atypical development as a result poor nutrition and diet.

fNIRS can be used in a wide age range of infants and children, allowing the potential for longitudinal studies of cognitive development from birth through infancy and into childhood. To date, there is a surprising lack of longitudinal data in the fNIRS literature however, in a separate study in Keneba we have gone on to demonstrate the use of fNIRS to assess cognitive function between 4–17 months in a longitudinal study and between birth and 24 months in a cross sectional study (unpublished observations). We believe that fNIRS is a viable tool with which to assess nutrition related cognitive development over the first few years of life in a resource poor setting, and that it may play an important role in informing targeted early intervention strategies. With this aim we have established a web-based portal for information relevant to global health researchers interested in optical imaging of cognitive development (www.globalfnirs.org).

## Methods

### Participants

Participants were identified from the West Kiang Demographic Surveillance System (http://www.ing.mrc.ac.uk/research_areas/west_kiang_dss.aspx). All infants were born full term (37–42 weeks gestation) and with normal birth weight. Exclusion criteria included weight-for-height or head circumference less than minus 3 z-scores against WHO standards. Growth measures indicate the infants were in the typical range for their age (see Table 1). Ethical approval was given by the joint Gambia Government - MRC Unit Ethics Committee, and written informed consent was obtained from all parents/carers prior to participation. Twenty four 4–8 month old infants participated in this study (10 female, mean age = 174.4 days, s.d. = 40.7). A further 18 infants participated but were excluded from the study due to an insufficient number of valid trials according to looking time measures (7 infants), experimenter error (7 infants), or due to tiredness/fussiness (4 infants). This attrition rate is within the standard range for infant fNIRS studies (see review by^8^).

### Experimental Procedures

**Figure 5:**
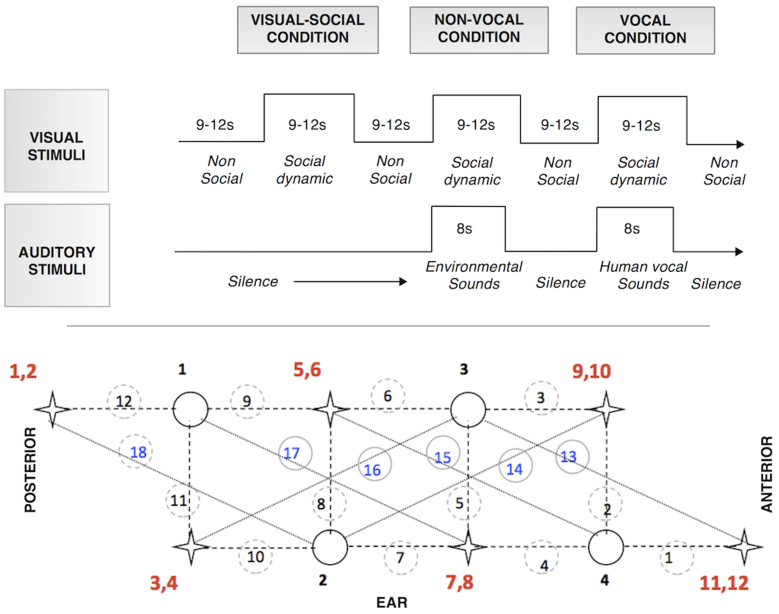
Illustrations of the procedure. Upper panel: The experimental design showing the order and timing of stimulus presentation for the three conditions (visual-social, vocal and non-vocal). Lower panel: The array design showing the location of the channels (dashed circle), sources (star), detectors (full circle), 2 cm (dashed line) and 4.5 cm (dotted line) channels. The headgear was placed over the right hemisphere with the source optode between channel 4 and 7 centred above the pre-auricular point (T4 according to the 10–20 system).

Infants wore custom-built fNIRS headgear consisting of an array over the right hemisphere (see Figure 5), containing a total of 18 channels (source-detector separations; 12 at 2 cm and 6 at 4.5 cm), and were tested with the UCL optical topography system^56^. Note that measurements were restricted to the right hemisphere as (1) our funding only allowed for a restricted number of sources and detectors with respect to the NIRS system used in the UK, and (2) we localized the channels to one hemisphere to ensure we could measure the full extent of the temporal lobe. This system uses near-infrared light of two different wavelengths (780 nm and 850 nm) to make spectroscopic measurements. Previously in studies in the UK we have noted the requirement to increase the source power levels of the NIRS system to ensure adequate signal to noise when studying infants with dark skin in which the increased levels of melanin result in stronger light attenuation. As all of the Gambian infants that we studied had dark skin we therefore used this increased source power level in all of our studies. The different channel separations allowed the measurement of activation at different depths into the cortex. Based on an understanding of light transport and given that the cortex is approximately 0.5 cm from the skin surface in this age group (measure taken from structural MRIs^57^; the channel separation used in the current study was predicted to penetrate up to a depth of approximately 2 cm from the skin surface, potentially allowing measurement of both the gyri and parts of the sulci near the surface of the cortex. Before the infants began the study, head measurements were taken to align the headgear with 10–20 coordinates^47^. Measurements from this group of infants showed that the average head circumference was 42.12 cm (s.d = 1.36). The headgear was placed over the right hemisphere with the source light optode between channel 4 and 7 centred above the pre-auricular point (T4 according to the 10–20 system). With the use of age-appropriate infant structural MRIs, anatomical scalp landmarks, and the 10–20 system, we can therefore approximate the location of underlying cortical regions for this group of infants.

In addition to the fNIRS study, anthropometric measures were performed. Infant lengths and weights were measured by using a Harpenden Infantometer length board (Holtain Ltd) and electronic baby scale (model 336; Seca), to a precision of 0.1 cm and 0.01 kg, respectively. Left-side triceps, biceps, and subscapular skinfold thicknesses were measured with a skinfold-thickness caliper (Holtain Tanner/Whitehouse) to 2-mm precision, and midupper arm circumference (MUAC) was measured by using a paper measuring tape to a precision of 0.1 cm. Head circumference, as a proxy for brain size, was measured to the nearest 0.1 cm with a stretch-proof measuring tape (model201; Seca) around the maximum circumference of the head (forehead to occiput).

Once the fNIRS headgear was placed on their heads, the infants sat on their parent's lap in front of a screen. The parent was instructed to refrain from interacting with the infant during the stimuli presentation unless the infant became fussy or sought their attention. The sequence of stimulus presentation has been used in previous research^22^,^47^. The conditions alternated one after the other, with a period of baseline between each (Figure 5). The three types of conditions (*visual-social (silent) V-S, auditory vocal V*, *auditory* *non-vocal N-V)* were presented in the same order across infants in a repeating loop (V-S, N-V, V, V-S, V, N-V) of trials (single presentation of a condition) until the infants became bored or fussy as judged by the experimenter who was monitoring their behavior. The reference for the haemodynamic change observed in response to the different conditions was obtained from the baseline (described in the following section). Therefore, the resulting activation was specific to the nature of the stimuli (or the contrast of two auditory stimuli) rather than visual or auditory stimulation per se.

A restriction of studying auditory processing in awake infants of this age is that they need to be presented with concurrent visual stimulation to reduce infant movement and thus artifact in the signal. We chose to employ the same visual stimuli during the presentation of the auditory stimuli bearing in mind that we collected data from the same stimulus without auditory stimulation.

### Visual stimuli

These consisted of full-color, life-size (head and shoulders only) social videos of adults (resident in The Gambia) who either moved their eyes left or right or performed hand games —“Peek-a-boo” and “Incy Wincy Spider.” Two visual social videos were presented for varying duration over each 9–12 s trial to avoid inducing anticipatory brain activity. To control for effects of attention - given that the social visual stimuli was sometimes presented simultaneously with auditory stimuli - there were six different visual social videos (two actors; three types of social video), while each auditory condition employed two different recordings (two speakers; one recording each – see below). During the baseline, visual stimuli were displayed, which consisted of full-color still images of different types of transport (i.e., cars and helicopters) presented randomly for a pseudorandom duration (1–3 s) for 9–12 s^22^. Dynamic non-social baseline stimuli have also been used in previous work investigating responses to visual social dynamic stimuli, and have been found to produce similar effects to the static non-social baseline used in the current study^48^,^51^. These visual stimuli were displayed on a 24-inch plasma screen with a viewing distance of approximately 100 cm.

### Auditory Stimuli

During the presentation of visual stimuli the infants were presented with auditory stimuli (see Figure 5) at the onset of two of every three of the trials. The content and duration of the social videos (9–12 s) were not synchronized with the auditory stimuli. Each auditory stimulus presentation lasted 8 s and consisted of four different sounds (of vocal or non-vocal stimuli) presented for 0.37–2.92 s each, interleaved by short silence periods (of 0.16–0.24 s). The two auditory conditions were equivalent in terms of average sound intensity and duration (p > .65). Within the vocal condition infants were presented with non-speech adult vocalizations of two speakers (who coughed, yawned, laughed, and cried). Within the non-vocal condition, the infants were presented with naturalistic environmental sounds (that were not human or animal produced, but were likely to be familiar to infants of this age; running water, bells and rattles). Vocal and non-vocal stimuli were chosen from the Montreal Affective Voices (for more detail, see^58^) and the stimuli of the voice functional localizer (http://vnl.psy.gla.ac.uk/resources_main.php). Additional non-vocal stimuli (toy sounds) were also recorded by the authors^1^.

### Data processing and analysis

Changes in HbO_2_ and HHb chromophore concentration (μmol) were calculated and used as hemodynamic indicators of neural activity^59^. Initially, the recorded near infrared attenuation measurements for each infant were analyzed, and trials, channels or participant data were rejected from further analysis by looking time measures (videos were coded offline by a researcher unfamiliar with the study's aims: >60% session looking considered valid) and the quality of the signals, using artifact detection algorithms^8^,^48^. For each infant, the trials and channels that survived these rejection criteria were entered into further analyses. Inclusion criteria required each channel to contain valid data in all three conditions. A minimum of three valid trials per condition was set as a threshold for inclusion within infants. Grand averaged time response curves of the hemodynamic responses (across all infants) for each channel were compiled. A time window was selected between 8 and 16 s post-stimulus onset for each trial. This period of time was selected to include the range of maximum concentration changes observed across infants for HbO_2_ and HHb, as illustrated by the haemodynamic time courses provided in the Supplementary materials. In an initial analysis, the grand averaged haemodynamic responses (µMol) of all infants were assessed for each of the three conditions (visual-social, vocal and non-vocal). For each channel, the maximum change (or amplitude) in HbO_2_ (increase in chromophore concentration) and/or HHb (decrease in chromophore concentration) in response to each experimental condition was assessed relative to baseline during the specified time window. Either a significant increase in HbO_2_ concentration, or a significant decrease in HHb, is commonly accepted as an indicator of cortical activation in infant work^8^. During statistical analyses, if HbO_2_ and HHb were either to increase or decrease significantly in unison, the signal was considered inconsistent with a haemodynamic response to functional activation^59^ and not reported in the analyses (for further discussion of physiological changes reported in infant fNIRS work see^8^,^13^). To resolve statistical problems of multiple comparisons for these group analyses we applied the false discovery rate (FDR) correction^60^. The channels that did not survive this correction are highlighted in Table 2.

## Supplementary Material

Supplementary Information Dataset 1

## Figures and Tables

**Table 1:** Growth measures (anthropometric scores) for the 4–8 month olds

Characteristics	
Sex (m/f)	10/14
Age (days)	174.4 ± 40.7^3^
Weight (kg)	6.91 ± 0.75
Length (cm)	64.46 ± 3.39
Head circumference H-C (cm)	41.45 ± 1.3
MUAC^1^ (cm)	13.8 ± 0.59
*Growth anthropometric z-scores*^2^	
Weight-for-age	−0.75 ± 0.86^4^
Length-for-age	−0.82 ± 1.21
H-C-for-age	−0.89 ± 0.99
Length-for-weight	−0.21 ±0.82

^1^Mid upper arm circumference;

^2^With the use of WHO reference curves.

^3^Mean ± SD (all such values).

^4^z score ± SD (all such values thereafter).

**Table 2:** The channel-by-channel t-test (two-tailed) analysis for the contrast between each of the experimental conditions (auditory: vocal and non-vocal; visual only) and the baseline (silence). For each contrast the results for the significant increase in HbO_2_ and/or decrease in HHb concentration are displayed. Channel tests that would not survive a False Discovery Rate analysis are highlighted (*)

*HbO_2_ (uM)*				*HHb (uM)*				*HbO_2_ (uM)*					*HHb (uM)*			
**Ch**	*t*	*p*	*df*	**Ch**	*t*	*p*	*df*	**Ch**		*t*	*p*	*df*	**Ch**	*t*	*p*	*df*
**Voice Condition vs Baseline**									**Non-Voice Condition vs Baseline**							
**5**	4.16	0.00038	*23*	**4**	3.02	0.006	*23*	2*		2.62	0.016	*23*	6*	2.23	0.0356	
**6**	3.08	0.00053	*23*	8*	2.42	0.024	*23*	**5**		3.60	0.0015	*23*	**9**	3.85	0.0008	
7*	3.88	0.044	23	**9**	3.46	0.002	*23*	**6**		3.83	0.00085	*23*	12*	2.37	0.026	
**8**	6.66	<0.00001	*23*	11*	2.08	0.049	*23*	7*		2.43	0.023	*23*				
**9**	5.30	0.00002	*23*	17*	7.46	0.018	6	**8**		9.36	<0.00001	*23*				
**11**	8.47	<0.00001	*23*					**9**		6.47	<0.00001	*23*				
**12**	5.40	0.000017	*23*					**11**		6.02	<0.00001	*23*				
15*	2.37	0.049	*9*					**12**		3.45	0.0022	*23*				
**16**	8.07	0.004	*7*													
**17**	8.48	0.0136	*6*													
**Visual Condition vs Baseline**									**Vocal > Non-Vocal Condition**							
9*	2.68	0.013	*23*										2*	2.09	0.049	*23*
**11**	3.39	0.0025	*23*										10*	2.15	0.042	23
**12**	3.89	0.00075	*23*										11*	2.29	0.031	23
**17**	7.49	0.0017	*8*													
18*	4.97	0.038	*6*													
