# Supplementary figures and images for: Functional near infrared spectroscopy (fNIRS) to assess cognitive function in infants in rural Africa
Source: Sci Rep. 2014 Apr 22;4:4740. doi: 10.1038/srep04740 (PMC5381189; doi:10.1038/srep04740)

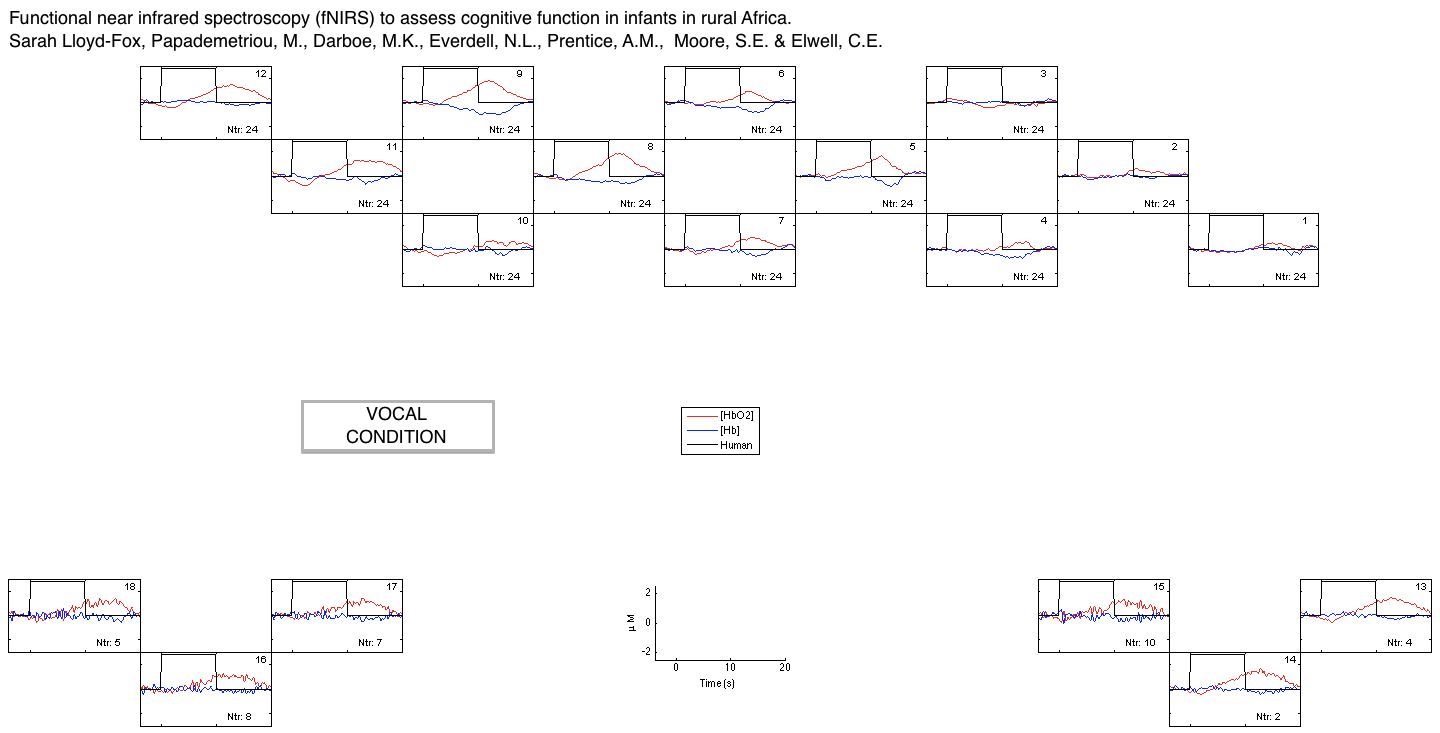

Supplement: Supplementary Information — Dataset 1 [file srep04740-s1.zip › Suppfig group plotstif.tif]
